# *HTRA1* Regulates Subclinical Inflammation and Activates Proangiogenic Response in the Retina and Choroid

**DOI:** 10.3390/ijms231810206

**Published:** 2022-09-06

**Authors:** Waseem Ahamed, Richard Ming Chuan Yu, Yang Pan, Takeshi Iwata, Veluchamy Amutha Barathi, Yeo Sia Wey, Sai Bo Bo Tun, Beiying Qiu, Alison Tan, Xiaomeng Wang, Chui Ming Gemmy Cheung, Tien Yin Wong, Yasuo Yanagi

**Affiliations:** 1 Singapore National Eye Centre, Singapore Eye Research Institute, 11 Third Hospital Ave, Singapore 168751, Singapore; 2 Molecular and Cellular Biology Division, National Institute of Sensory Organs, National Hospital Organization Tokyo Medical Center, Tokyo 152-8902, Japan; 3 Academic Clinical Program, Duke-NUS Medical School, National University of Singapore, Singapore 169857, Singapore; 4 Institute of Molecular and Cell Biology, Agency for Science, Technology and Research (A*STAR), Singapore 138673, Singapore

**Keywords:** HtrA1 mice, age-related macular degeneration, polypoidal choroidal neovascularization, RNA-sequencing, subclinical inflammation, proangiogenic response, KEGG & Reactome pathways, immune system process GO-terms

## Abstract

High-temperature requirement A1 (HtrA1) has been identified as a disease-susceptibility gene for age-related macular degeneration (AMD) including polypoidal choroidal neovasculopathy (PCV). We characterized the underlying phenotypic changes of transgenic (Tg) mice expressing ubiquitous CAG promoter (CAG-HtrA1 Tg). In vivo imaging modalities and histopathology were performed to investigate the possible neovascularization, drusen formation, and infiltration of macrophages. Subretinal white material deposition and scattered white-yellowish retinal foci were detected on CFP [(Tg—33% (20/60) and wild-type (WT)—7% (1/15), *p* < 0.05]. In 40% (4/10) of the CAG-HtrA1 Tg retina, ICGA showed punctate hyperfluorescent spots. There was no leakage on FFA and OCTA failed to confirm vascular flow signals from the subretinal materials. Increased macrophages and RPE cell migrations were noted from histopathological sections. Monocyte subpopulations were increased in peripheral blood in the CAG-HtrA1 Tg mice (*p* < 0.05). Laser induced CNV in the CAG-HtrA1 Tg mice and showed increased leakage from CNV compared to WT mice (*p* < 0.05). Finally, choroidal explants of the old CAG-HtrA1 Tg mice demonstrated an increased area of sprouting (*p* < 0.05). Signs of subclinical inflammation was observed in CAG-HtrA1 Tg mice. Such subclinical inflammation may have resulted in increased RPE cell activation and angiogenic potential.

## 1. Introduction

Age-related macular degeneration (AMD) is a progressive eye disease and a leading cause of severe central vision loss in elderly people aged over 65 in developed countries [1]. Acute loss of vision occurs in neovascular AMD (nAMD), i.e., an advanced stage of AMD, with the development of macular neovascularization (MNV). Polypoidal choroidal vasculopathy (PCV) is a common subtype of neovascular AMD and type 1 neovascularization (NV) variant in Asians. Polypoidal lesions and branching neovascular networks are the hallmarks of PCV [2]. Although PCV shares some phenotypical features similar to those of neovascular AMD, such as exudation, hemorrhages, scarring, and fibrosis, its clinical manifestations and treatment outcomes are fairly different [3].

Genome-wide association studies reported that HtrA1 polymorphism was linked to neovascular AMD and PCV in Asian populations [4,5,6]. HtrA1 is highly expressed in the retinal pigment epithelium (RPE), drusen, and abnormal choroidal vasculature [7]. Importantly, although there is some debate [8], the risk polymorphism at the promoter region and a unique insertion/deletion sequence located upstream of HtrA1 were suggested to be related to overexpression of the gene among nAMD patients [6,9]. Subsequent studies using mouse models overexpressing HtrA1 demonstrated that it plays an important role in the pathogenesis of AMD. First, degradation of elastin and extracellular matrix proteins were noted in the Bruch’s membrane and choroidal vessels in mice overexpressing HtrA1 [7,10,11,12]. Second, some transgenic mice lines overexpressing HtrA1 reportedly developed spontaneous choroidal neovascularization (CNV), including PCV. Interestingly, HtrA1 transgenic (Tg) mouse models using ubiquitous CAG promotor developed spontaneous CNV [13], whereas another group used RPE-specific promotor with overexpressed human HtrA1 in RPE cells, and showed PCV phenotypes [7]. Angiographically, CNV and PCV show some overlapping but somewhat distinct phenotypes and the question remains whether the difference in the phenotypes between both groups could be attributable to the differences in promoters used in these mouse lines. Although the main differences in angiogenic process between CNV and PCV remain unknown, some of the earliest events in both CNV and PCV are considered to start with alterations in the RPE and Bruch’s membrane [14].

In vivo mouse imaging forms an essential diagnostic tool for retinal and choroidal vasculature, giving detailed information on retinal morphology, leakages, and perfusion. The recent advent of mouse optical coherence tomography angiography (OCTA), among others, allows three-dimensional view of the retina and choroid. With its blood motion contrast feature, OCTA offers a convenient method to investigate the vascularity status of corresponding lesions [15]. Though aforementioned studies [10,16], demonstrated PCV phenotypes through in vivo imaging, they mainly relied on indocyanine green angiography (ICGA) to detect polypoidal lesions and no detailed phenotypical analysis using OCTA was performed on the retino-choroidal lesions of HtrA1 Tg mice so far. In this regard, it is worth mentioning that the interpretation of ICGA is sometimes not straightforward and the lesions that appear as polypoidal lesions are not necessarily those observed in PCV, as pointed out clinically [17].

We hypothesized that a detailed phenotyping study using multiple in vivo imaging modalities would facilitate our understanding of the role of HtrA1 in AMD/PCV. In this study, we performed in vivo imaging with the use of the detailed multimodal imaging analysis of the lesions, together with histopathological studies on the CAG-HtrA1 Tg mice to address the pathological changes. Furthermore, in vivo and ex vivo neovascularization assays were conducted using laser-induced CNV and a choroidal sprouting assay to clarify the angiogenic function of HtrA1.

## 2. Results

### 2.1. In Vivo Retinal Imaging of CAG-HtrA1 Tg Mice

**Figure 1:**
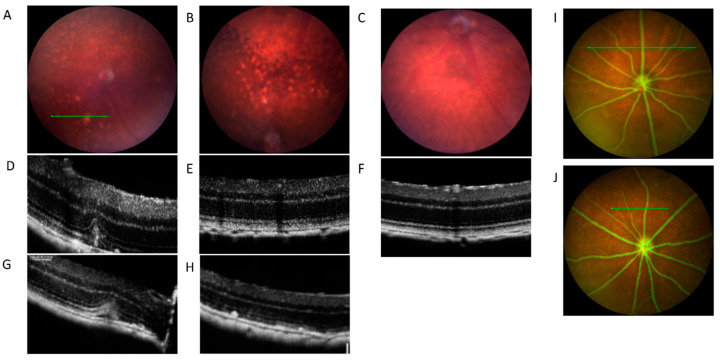
In vivo retinal imaging using combined color photography and OCT retinal imaging compared between CAG-HtrA1 Tg mice and wild-type (20 months). (**A**) CFP from the CAG-HtrA1 Tg mice shows the area of large white lesion accumulation (**B**) Yellow-whitish foci scattered through the retina in the CAG-HtrA1 Tg mice. (**C**,**F**) CFP and cross-sectional OCT scan from the wild type mice. (**D**,**G**) Cross-sectional OCT scans show the RPE elevation on OCT corresponding to the large white lesions, indicating the small amount of subretinal fluid. (**E**,**H**) Cross-sectional OCT showing diffuse irregular reflectivity of the RPE layer and ellipsoid zones (**E**) and some intraretinal hyper-reflective foci (**H**) in the eyes with yellow-whitish foci (**I**,**J**) No leakage, oozing, or staining from neither of the mice groups noted from the FFA images corresponding the lesion from the fundus.

Firstly, we investigated the color fundus photography (CFP), optical coherence tomography (OCT), and fundus fluorescein angiography (FFA) images for 20-month-old CAG-HtrA1 Tg mice and wild type (WT) mice (Figure 1). CFP showed numerous white lesions of various sizes scattered throughout entire fundus. Most of the lesions were smaller than 0.5-disc area, but in a typical eye, one or two lesions were larger than 0.5-disc area. Of the 60 CAG-HtrA1 Tg mice we imaged using OCT, 20 of them exhibited large (i.e., 20~50% disc diameter) white abnormal lesions ((Tg—33% (20/60) and wild-type (WT)~7% (1/15), *p* < 0.05), and these lesions were present only in one mouse in the wild-type control (1/15; *p* = 0.04 with Chi-Square test). Based on multimodal imaging, the larger and smaller lesions seemed to have different features, as such we designated the former and latter lesions as type 1 and type 2 lesions, respectively. The characteristics of each lesion type are shown in Table 1. OCT corresponding to the area of type 1 lesion accumulation showed subretinal empty space presumably reflecting localized subretinal fluid (Figure 1A,D,G). These lesions were then studied extensively for retinal vascular changes via FFA. However, the lesions were neither hyper- nor hypo-fluorescent in the FFA images. The RPE layer and ellipsoid zones showed diffuse irregular reflectivity and some intraretinal hyper-reflective foci (Figure 1B,E,H), but there were no remarkable findings corresponding to type 2 lesions on cross sectional OCT.

**Figure 2:**
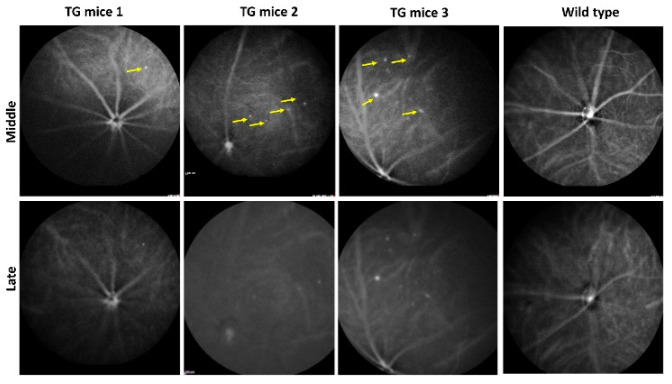
ICGA of CAG-HtrA1 and wild-type mice. Time course images shows middle to late phase ICGA features in the CAG-HtrA1 Tg mice and wild-type control. Punctate hyperfluorescent spots (yellow arrows) appears in the middle phase ICGA (can be observed in the Tg mice while no notable lesions in the wild-type mice. These lesions are considered as inner choroidal spots.

**Figure 3:**
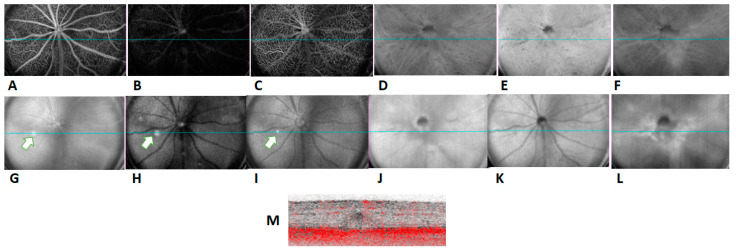
In vivo imaging of mice retina and choroidal plexus. Representative angiographic (**A**–**F**) and corresponding structural (**G**–**L**) images from HtrA1 mice (27 months) of OCTA. Images from the superficial plexus slab (**A**,**G**), avascular slab (**B**,**H**), deep inner plexus slab (**C**,**I**), choriocapillaris slab (**D**,**J**), ORCC slab (**E**,**K**), and the choroid slab (**F**,**L**). Image of deep retinal plexus (**I**) indicates the selected area (white arrow) of the cross B section scan image with flow signal in red (**M**). B scan failed to show vascularization corresponding to the Type-1 lesion (**G**–**I**).

We then used ICGA, a gold standard for the diagnosis of PCV in humans [18], to investigate if any polypoidal lesions were present in the CAG-HtrA1 Tg mice. ICGA was performed on 10 Tg mice which had at least one type 1 lesion featured in CFP and OCT, which did not reveal any vascular dilatation or branching neovascular networks in the Tg mice model or the WT. However, in the CAG-HtrA1 Tg mice multiple small punctate hyperfluorescent lesions were observed in the mid and late phase ICGA (Figure 2), which does not correspond to the type 1 or type 2 lesions. These punctate hyperfluorescent spots were not detectable on OCT or OCTA (Figure 3G,M, and we assume these are inner choroidal lesions. In addition, there was no flow signal from the type 1 lesions on cross sectional or en face OCTA images.

**Figure 4:**
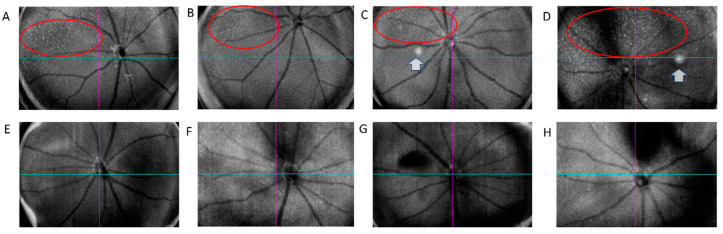
Representative OCT structural avascular plexus images of aged CAG-HtrA1 Tg and wild-type mice (26–29 months). Subretinal deposits (red ovals), which partially correspond to small white-yellowish retinal foci on CFP, were scattered through the avascular slab while wild-type shows minimal deposits. Arrows indicate large white lesions (type 1 lesions). Representative photographs from four mice per group are shown (**A**–**D**): CAG-HtrA1 Tg and (**E**–**H**): Wt.

In contrast to cross-sectional OCT, en face wide-field OCT confirmed numerous small subretinal deposits at the level of “avascular” slab (comprised from the outer boundary of the outer plexiform layer to 99 µm above the RPE (Figure 3) throughout the entire retina in the CAG-HtrA1 Tg mice compared to the wild-type control (Figure 4), which partially overlapped with type 2 lesions.

### 2.2. Increased Macrophage Infiltration and Activation in CAG-HtrA1 Tg Mice Retina

**Figure 5:**
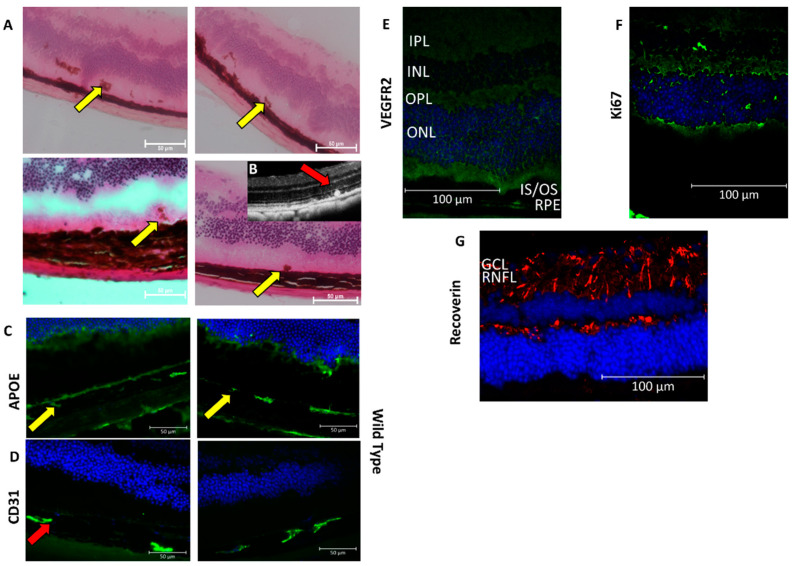
Immunohistochemical analysis of the retina from CAG-HtrA1 Tg mice vs. wild-type control (17 months). (**A**) Hematoxylin and eosin staining of the whole retina of CAG-HtrA1 Tg shows the flecked structures (yellow arrows) in the RPE layer denoting the presence of RPE migration in the retina. (**B**) RPE migration reflected in the OCT corresponding to the H&E stains (**C**,**D**) showing expression of ApoE (yellow arrows) in the RPE and CD31 in the retina and the choroid (red arrows) confirming that there is no significant difference between the CAG-HtrA1 Tg mice and wild-type mice groups. (**F**) Ki67, proliferation marker was not detected in CAG-HtrA1 Tg mice. (**E**) VEGFR2 and (**G**) Recoverin showed positive. Nuclei were stained with DAPI (blue). (Abbreviations: RNFL: retinal nerve fibre layer; GCL: ganglion cell layer; IPL: inner plexiform layer; INL: inner nuclear layer; OPL: outer plexiform layer; ONL: outer nuclear layer; IS/OS: inner and outer segment; and RPE: retinal pigment epithelium).

With the H&E staining, infiltration of melanin-containing cells was observed in the subretinal space and the outer retina, corroborating the observation of small pre-RPE hyper-reflective foci on cross-sectional OCT, which is suggestive of RPE migration in the CAG-HtrA1 Tg mice (Figure 5A,B). Histological analysis demonstrated that ApoE, a component for drusen, Recoverin, a photoreceptor marker, CD31 and VEGFR2, endothelial markers, were expressed in both groups at 17 months (*n* = 2 for both Tg and WT) (Figure 5C–E,G). However, there was no difference in the expression pattern of these markers between the groups, which suggests that it is a common phenomenon of an age-related change in the eye.

**Figure 6:**
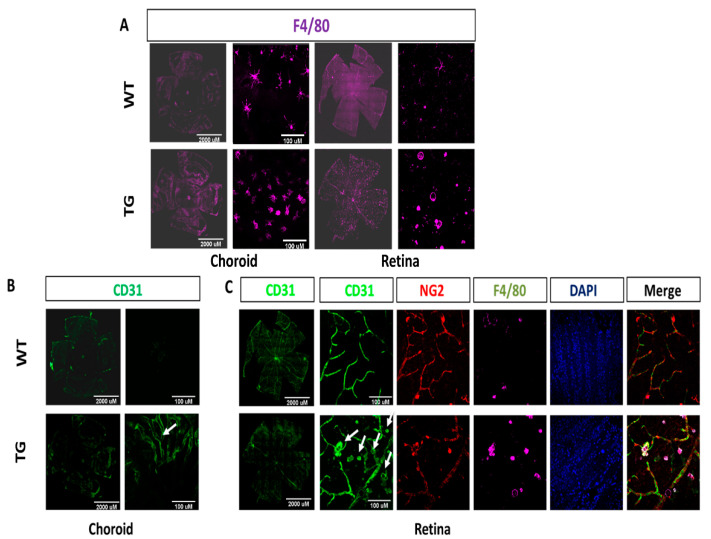
Macrophage infiltration and activation in subretina and retina of CAG-HtrA1 Tg mice compared to wild-type control. Representative images of flat mount of retina and choroid separated from CAG-HtrA1 Tg mice (27 months) and wild-type control (29 months). (**A**) F4/80 (macrophage infiltration) (**B**) torturous choroidal vessels (white arrows) were observed in HtrA1 Tg mice. (**C**) Multicolor immunofluorescence showing CD31 positive endothelial cells, NG2 positive pericytes, and F4/80 positive macrophages. F4/80+ and CD31+ double-positive cells are present in the CAG-HtrA1 Tg mice, and some CD31 + F4/80+ cells co-express NG2, a well-accepted pericyte marker, in the CAG-HtrA1 Tg mice. Coverage of NG2 pericyte onto CD31+ vessels was also decreased in the CAG-HtrA1 Tg mice.

Furthermore, to investigate the level of macrophage infiltration between both groups, we stained the retina flatmounts with NG2, CD31, and F4/80 antibodies. The results demonstrated that the number of F4/80 positive macrophages in the subretinal space and the outer retina of CAG-HtrA1 Tg mice was numerically higher compared to that in the wild-type controls (308 (142) vs. 197 (71) per eye [mean (SD)] for Tg (*n* = 9) and WT (*n* = 3), respectively *p* = 0.232, Figure 6A). Additionally, tortuous choroidal vessels were observed in 28-month-old CAG-HtrA1 Tg mice (Figure 6B). Moreover, multicolor immunofluorescence revealed the presence of CD31 + F4/80+ double-positive cells in the CAG-HtrA1 Tg mice, and that some CD31 + F4/80+ cells co-express NG2, a well-accepted pericyte marker, suggesting the presence of mature activated CD31 + F4/80+ macrophages transdifferentiating into defined pericytes. Coverage of NG2 pericyte onto CD31+ vessels was also decreased in the CAG-HtrA1 Tg mice (Figure 6C).

### 2.3. Increased Peripheral Blood Monocyte Subsets in CAG-HtrA1 Tg Mice

**Figure 7:**
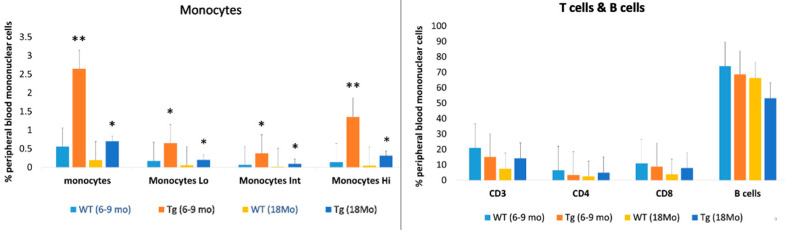
Comparison of monocytes, T cells, and B cells in CAG-HtrA1 Tg mice and wild-type control aged between 6–9 months and 18 months. Monocytes are significantly higher in the Tg mice while there is no significant difference in the T cells and B cells between the mice groups. Tg and WT mice. (*n* = 4 each) were used for the comparison of population of granulocytes including monocytes and the Mann–Whitney U test was performed. Significance level was defined as ** *p* < 0.01 and * *p* < 0.05.

Since CD31 + F4/80+ cells are also found in circulating monocytes [19], we then asked whether monocytes subpopulations were different between CAG-HtrA1 Tg mice and age-match controls. Peripheral blood cells (PBMC) were obtained from CAG-HtrA1 Tg mice and age-match controls. Monocytes subpopulations were compared between both mice groups aged 6–9 months and 18 months. CAG-HtrA1 Tg mice showed a significantly higher number of all monocyte subpopulations (classical, intermediate, and non-classical) in both at 6–9 months and 18 months (Figure 7). We also tested the T cells and B cells in the mice of both groups; however, there was no difference between the groups.

### 2.4. Increased Choroidal Angiogenic Potential of CAG-HtrA1 Tg Mice

**Figure 8:**
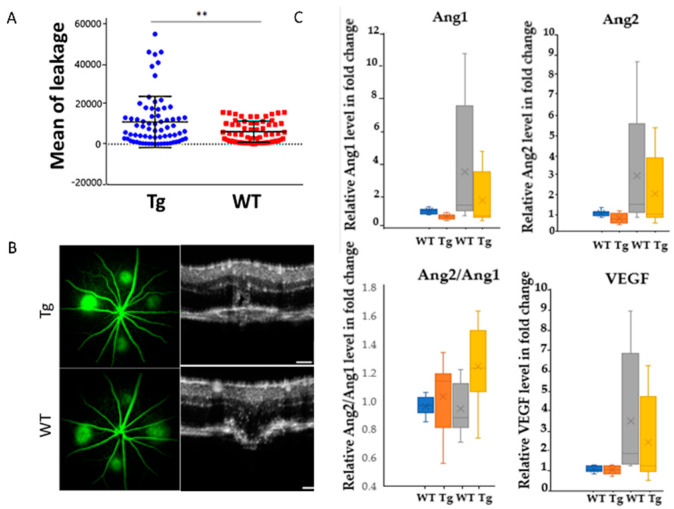
Leakage and change of angiogenic factors expression in laser-induced CNV mice. (**A**) Dye leakage from the CNV lesion in CAG-HtrA1 Tg mice and wild-type control (10 weeks) after one-week observation post laser treatment. Mean leakage is calculated between the CAG-HtrA1 Tg mice and wild-type control. (**B**) Representative FFA image and the corresponding lesions of the OCT. (**C**) Multiplex assay. Expression level of cytokine Ang1 is significantly decreased in CAG-HtrA1 Tg compared to WT (**Left**). After laser treatment, Ang1, Ang2, and VEGF were upregulated. The ratio of Ang2/Ang1 was significantly increased in CAG- HtrA1Tg mice after laser treatment. 10 weeks, (*n* = 8) for each group (pre- and post-laser). The Mann-Whitney U test was performed. Significance level was defined as ** *p* < 0.01. (NT: no treatment, WT: wild type, Tg: CAG-HtrA1 Tg).

We next investigated how HtrA1 overexpression was linked to angiogenesis. In vivo laser photocoagulation was performed to induce CNV lesions in mice (8–10 weeks). The area of fluorescein leakage as determined by FFA was significantly increased in the CAG-HtrA1 Tg mice compared to that in wild-type controls, suggesting that overexpression of HtrA1 gene may promote the CNV (Figure 8A,B). Next, the multiplex analysis demonstrated that among angiogenic factors investigated, the basal expression levels of Angiopoietin-1 (Ang1) were lower in CAG-HtrA1 Tg mice compared to control wild-type mice (Figure 8C). Three days after the laser treatment, Ang1, Angiopoietin-2 (Ang2), and Vascular endothelial growth factor (VEGF) were upregulated. Of note, the ratio of Ang2/Ang1 was significantly increased in CAG-HtrA1Tg mice after laser treatment. 

**Figure 9:**
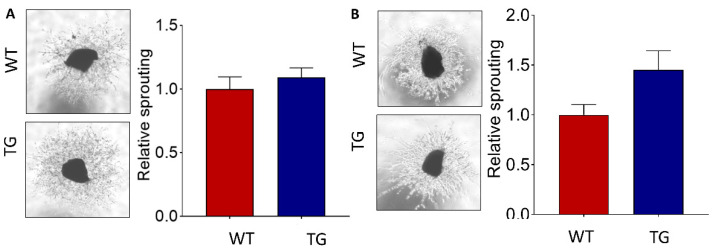
HtrA1 increased choroidal angiogenic potential of CAG-HtrA1 Tg mice. The choroid sprouting assay enables analysis of the relationship between choroidal endothelial cells and RPE allowing for the discovery of mechanisms that control choroidal vascularity to sub-retinal proliferative disorders. (**A**,**B**) Representative images and quantitative analysis of vessel outgrowth from P3 choroid from P3 mice (*n* = 3) and 52-week-old mice (*n* = 2) of CAG-HtrA1 Tg and wild-type controls. Although there was no difference in the choroidal sprouting using the postnatal day 3 choroidal explants, choroidal explants from aged CAG-HtrA1 Tg mice showed an increased relative sprouting area than the wild-type mice. ImageJ software was used to quantify this assay by counting the number and length of sprouting vessels.

Lastly, ex vivo, choroid sprouting assay [20], was carried out to further investigate the impact of HtrA1 overexpression in CNV. Choroid explants were isolated from postnatal day 3 (P3) and 52-week-old CAG-HtrA1 Tg and age-matched wild-type control mice. We first observed the impact of age factor on choroidal vascular proliferation in CAG-HtrA1 Tg mice and wild-type control mice; explants from aged mice are more angiogenic than those from the P3 mice in both groups. Comparison between CAG-HtrA1 Tg and wild-type mice showed that although there was no difference in the choroidal sprouting using the postnatal day 3 choroidal explants, choroidal explants from aged CAG-HtrA1 Tg mice showed an increased relative sprouting area than the wild-type mice (Figure 9).

### 2.5. Reactome and KEGG Pathway Enrichment Analysis

**Figure 10:**
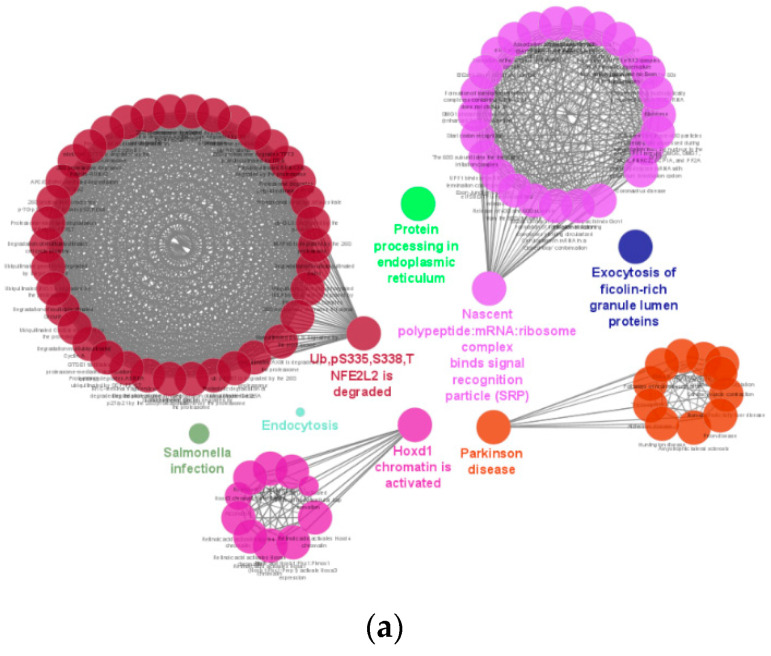

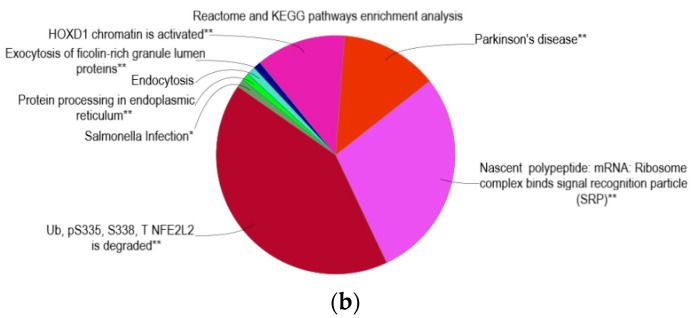
Signal interactions and pathway enrichment analysis. (**a**) DEGs of Tg versus WT were analyzed to identify the Reactome and KEGG pathways in which they involved by using Cytoscape with ClueGo. A total of seven significant identified pathway clusters were labelled in colored letters. A representative pathway for each cluster was selected by the percentage of genes involved per term as well as the number of genes matched in the term. (**b**) Pie chart of identified pathways based on Reactome and KEGG pathways. Seven significant pathways (group *p* value corrected with Bonferroni step down) were marked with asterisk (** for *p* < 0.01 and * for *p* < 0.05) among eight total pathways.

Pathways enrichment analysis of all DEGs from Tg vs. WT was conducted by using the Cytoscape with ClueGo. Reactome and KEGG pathways data were selected as reference resources. A total of eight clusters were identified. One representative pathway was selected from each cluster based on percentage of association to the pathway as well as total number of genes involved. Identified eight enriched pathways were (1) HOXD1 chromatin is activated, (2) Parkinson’s disease, (3) Complex of nascent polypeptide: mRNA: ribosome binds signal recognition particle (SRP), (4) Ub. pS335, S338, T NFE2L2 is degraded, (5) Salmonella infection, (6) Protein processing in endoplasmic reticulum, (7) Endocytosis, (8) Exocytosis of ficolin-rich granule lumen proteins (Figure 10a,b). All pathways were found to be significant except the endocytosis pathway.

### 2.6. Immune System Network and GO-Terms Pathway Enrichment Analysis

**Figure 11:**
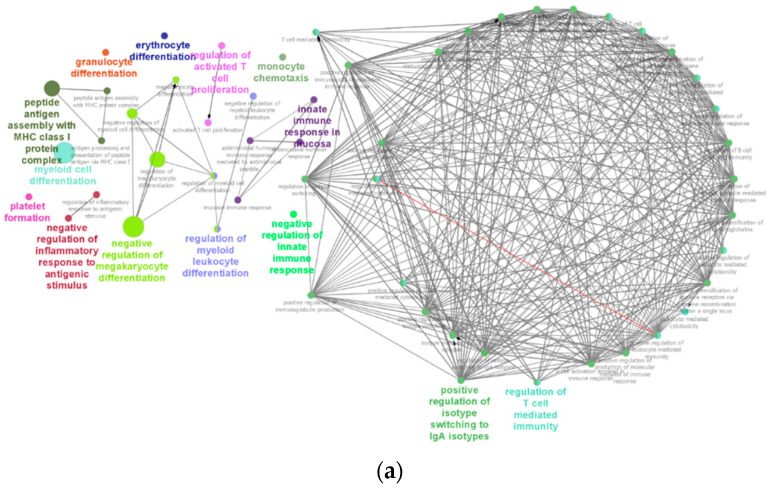

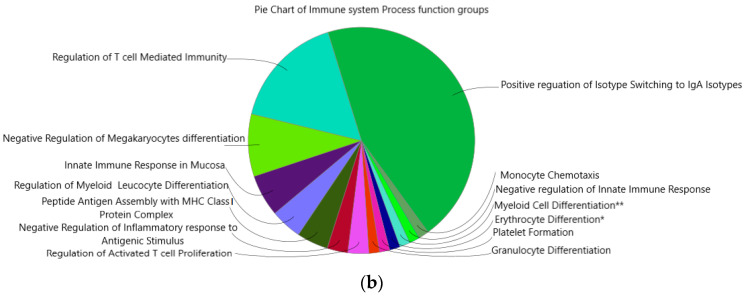
Networks of immune system process of gene ontology (GO) of DEGs in Tg mice. (**a**) Significant differentially expressed genes from RNA-seq analysis of Tg versus WT mice (*n* = 8 each) were further analyzed by Cytoscape software with ClueGo to visualize immune system related molecular level interaction networks that were over-represented in Tg mice. The larger the node size, the smaller the GO-terms *p* value is reflected. Involvement of a number of common genes between the nodes are represented by the degree of the thickness of the lines. Pointed arrowhead indicates activation/positively regulation. Diamond arrowhead indicates regulation. Immune system process of GO-terms for each group is labelled in colored bold letters. (**b**) A pie chart of immune system process GO-terms pathway network groups. A total of fourteen functional immune system process GO-terms groups were involved more prominently in Tg mice compared to WT mice. Significant networks of the immune system (group *p* value corrected with Bonferroni step down) were indicated by asterisk (** for *p* < 0.01 and * for *p* < 0.05).

Functional groups of immune system process GO-terms and pathway enrichment were identified by using the Cytoscape software with ClueGo. Tg mice showed over-represented fourteen immune systems process GO-terms and networks (Figure 11a,b): (1) positive regulation of isotype switching to IgA isotype, (2) regulation of T-cell mediated immunity, (3) negative regulation of megakaryocytes differentiation, (4) innate immune response in mucosa, (5) regulation of myeloid leucocyte differentiation, (6) peptide antigen assembly with MHC class protein complex, (7) negative regulation of inflammatory response to antigenic stimulus, (8) regulation of activated T cell proliferation, (9) monocyte chemotaxis, (10) negative regulation of innate immune response, (11) myeloid cell differentiation, (12) Erythrocyte differentiation, (13) platelet formation, (14) granulocyte differentiation. Immune system networks such as myeloid cell differentiation and erythrocyte differentiation were found to be significant among other immune system enrichment pathways.

## 3. Discussion

### 3.1. Key Findings in the Current Study

The first objective of this study was to carry out a detailed phenotypic analysis and describe its morphologic and vascular changes in the CAG-HtrA1 Tg mice. Using several in vivo imaging modalities, specifically OCT and OCTA, we accurately obtained non-invasive deep morphological images of the retinal and choroidal layers together with their flow signals, suggestive of the vasculature, through tuneable lasers [15,21,22]. On OCT, CAG-HtrA1 Tg mice showed three-dimensional morphological changes of the yellow-white retinal foci and large white lesions. We detected two types of lesions in the retina. Type 1 lesions are larger (typically larger than 0.5-disc area), and located anterior to RPE, accompanied by localized subretinal fluid on OCT. Type 2 lesions are small foci on CFP that are not clearly visible on structural OCT, but appeared as pre-RPE material on en face OCT, and showed no staining on ICGA. FFA showed no leakages corresponding to the yellow-white foci and large white lesions (Figure 1). From the ICGA images in the choroidal region, punctate hyperfluorescent spots started to appear in the middle phase to the late phase.

### 3.2. Type 1 Lesions in the CAG-HtrA1 Tg Mice Were Different from Those in Human PCV

Flow signals corresponding to the type 1 large white deposits were not detected by OCTA [23]. Moreover, in the cross-sectional OCTA, there was no blood flow corresponding to the area of the type-1 lesion, suggesting that these lesions were not vascular. Accumulation of RPE/pre-RPE deposits (type-2 lesions) was scattered in the avascular plexus in the CAG-HtrA1 Tg mice compared to that in the wild-types. We speculate these retinal changes were in normal age-related processes but were accelerated in the CAG-HtrA1 Tg mice.

Recent clinical observations support that most eyes with PCV manifest features consistent with “pachychoroid”, which is characterized by thickened choroid, dilatation of choroidal vessels, and attenuation of choriocapillaris, and inner choroidal ischemia due to such choroidal changes likely contributes to the development of choroidal neovascularization [24]. In the current mouse model, there was no apparent abnormalities in the choroid except for punctate hyperfluorescent spots on ICGA, which presumably corresponds to punctate hyperfluorescent spots typically seen in pachychoid spectrum diseases [25,26], and somewhat tortuous choroidal vessels. As such, there may be subclinical choroidal functional changes in the CAG-HtrA1 Tg; however, there was no choroidal vascular hyperpermeability on ICGA. The choroidal structure of mice and humans are considerably different, and the current mouse model may not be a good model to study the pathogenesis of human choroidal diseases.

### 3.3. Activated Macrophages in the Subretinal Lesions and Increased Subclinical Inflammation

Regarding the yellow-white foci throughout the retina, it should be noted that the RPE/pre-RPE deposits observed in the CAG-HtrA1 Tg mice were not drusen. We did not detect any differences in the expression pattern of ApoE, a lipid transporter which has a core function in the drusen formation and accumulates in RPE cells near drusen or in drusen itself [27,28,29]. In addition to that, there were no defects in the RPE corresponding to the subretinal foci. RNA-seq data analysis showed no differences in ApoE and vitronectin expression too. (Data not shown). In this regard, it is important to take note of the pathological differences between the human and mice phenotypes. Notably, the absence of macula in mice and its inability to develop deposits at the base of the RPE, like that of humans, forms a difference in the transportation of lipids across RPE [29]. Such yellow-whitish foci were sometimes reported in aged wild-type mice, and reportedly formed due to an age-dependent accumulation of microglia at the subretinal space [30]. Moreover, retina and choroidal flatmounts also suggested that there was increased macrophage infiltration and activation in the subretinal area in the CAG-HtrA1 Tg mice in the current study.

H&E images of the CAG-HtrA1 Tg mice showed that the lesions were shown to relate to RPE migrations. There are several possible theories of RPE migration. In the context of proliferative vitreoretinopathy, which is characterized by epithelio-mesenchymal transition of RPE cells followed by membrane growth and fibrosis; it is generally agreed that chemical factors in the vitreous, including TGF-beta, contribute to RPE cell proliferation and migration [31]. In the context of AMD, RPE activation and migration plays an important role for the development of atrophy [32], and presumably for neovascularization [33]. The RPE cells detach from the RPE basal lamina and subsequently migrates into the retina. A potential stressor for this event is chronic inflammation occurring in Bruch’s membrane [8,34,35]. A previous animal study suggested age-dependent accumulation of subretinal phagocytes similar to the current study [30]. A recent study in vitro also reported that overexpression of HtrA1 promoted proliferation of RPE cells [36,37]. Hence, considering all the factors, we postulated that RPE migration was due to the consequences of subclinical inflammation [38]. Although there was no shift towards an immature myeloid profile, there was an increase of all subsets of monocytes in the CAG-HtrA1 Tg mice, suggesting that there was a subclinical immune system activation, which might have also contributed the phenotypical change of retina in the CAG-HtrA1 Tg mice.

### 3.4. HtrA1-Induced Subclinical Inflammation and Pro-Angiogenic Activities

Laser-induced CNV model demonstrated that CAG-HtrA1 Tg mice are more susceptible to the development of laser-induced CNV compared to wild-type control mice. Our results suggest that HtrA1 overexpression may affect the angiopoietin/Tie2 signaling pathway; under inflammatory conditions, Ang2 is considered to act as an antagonist of the Tie2 receptor, and destabilizes the vascular endothelial cells [39]. Moreover, choroidal explants [20], from aged, but not postnatal CAG-HtrA1 Tg mice had more potent angiogenic properties, as shown in the relative sprouting area compared to the wild-type control, suggesting that not increased HtrA1 activity alone, but resultant age-dependent humoral and cellular changes contribute to angiogenesis [17]. Interestingly, HtrA1 is shown to be upregulated in the peripheral blood of retinopathy of prematurity (ROP) patients, a condition characterized by aberrant retinal vascular maturation and oxygen-induced retinopathy murine model of ROP demonstrated that mice overexpressing HtrA1 demonstrated greater ROP disease activity, supporting the pro-angiogenic function of HtrA1 [40]. Together, these results evidently support the phenomenon that the inflammatory milieu induced by the overexpressed HtrA1 possibly contributes to increased angiogenic response.

### 3.5. Enrichment of Homeobox D1 and Parkinson’s Disease Pathways

Homeobox (HOX) genes are responsible for embryogenesis, morphogenesis, tumorigenesis and cell differentiation [41,42]. Hitherto, four chromosomal clusters of the HOX family, HOXA, HOXB, HOXC, and HOXD, were reported. A prominent expression level of homeobox D1 (HOXD1) was detected in endothelial cells (ECs) and displayed its regulatory role in angiogenesis. This was evident when knocking HOXD1 out of human umbilical vein endothelial cells (HUVEC) showed inhibition of tube formation as well as endothelial cell migration. HOXD1 played critical role in the regulation of adhesion between ECs and extra cellular matrix by lowering the expression level of an adhesion molecule, integrin subunit beta 1 (ITGB1). The existence of the binding site of HOXD1 was found in the promotor region of ITGB1 in ECs and thus, the transcription of ITGB1 was also controlled by HOXD1 [43]. Furthermore, higher expression level of HOXD1 was discovered in blood-derived outgrowth endothelial cells (BOECs) along with HOXD3, -D4, -D8, and -D9 [44]. In our study, activation of HOXD1 chromatin was significant in Tg mice compared with WT mice. This RNA-seq result reflected that Tg mice showed more angiogenic phenotype compared to WT mice. Besides, other HOX chromatins, such as HOXD3, HOXC4, HOXB1, HOXB4, HOXA1, and HOXA4 were also activated. HOXD3 has been known to play a critical role in angiogenesis by inducing integrin beta-3 (ITGB3). In vivo, inducing angiogenesis studies in the skin of mice, in the membrane of chicken embryo chorioallantois, and in the brain of mice demonstrated the impact of HOXD3. Significant increased microvascular density was observed in the wounds of treated mice with HOXD3. Endotheliomas were found in the HOXD3-infected chick embryos. A significant increase in microvessels, especially cerebral angiogenesis, was observed in mice after the retrovirus-mediated HOXD3 gene was transferred into the mice brains. In their studies, the adhesion molecule, ITGB3 expression was induced significantly [45,46,47]. An association between angiogenesis and Parkinson’s disease (PD) was observed in both animal model as well as in human patients. A significant increase in endothelial cell count together with a higher level of expression of angiogenic factors, such as ITGB3 and VEGF, has been reported in the previous studies [48,49,50]. In our study, RNA-seq data analysis revealed that Tg mice showed enrichment of the PD pathway. Interestingly, pathway enrichment analysis of RNA-seq data of HtrA1 Tg mice showed two significant enriched pathways, ~ HOXD1 and Parkinson’s disease, that are evidently related to angiogenesis as discussed. Therefore, angiogenic phenotype was confirmed in our HtrA1 Tg mice used in this study.

### 3.6. Enrichment of IgA Isotype and Myeloid Cells Differentiation Pathways

RNA-seq data were further analyzed to view functional immune system process GO-terms. IgA and antibody secreting plasmablast cells have been found to be significantly elevated in the serum and tears of age-related macular degeneration (AMD) subjects. Elevation of this antibody was specific to the IgA isotype since no other antibodies, IgM, or IgG level were changed significantly. Furthermore, it was reported that a higher titer of IgA was directly proportional to the stage of AMD. In the experiment, peripheral blood and tears of 20 AMD subjects (10 each of early and late AMD) and 15 healthy control subjects were investigated [51]. Furthermore, nowadays, IgA has been known to be involved in the initiation of inflammation not only in mucosal sites but also in non-mucosal sites. IgA is crucial in the production of proinflammatory cytokines from myeloid cells by activating its FC receptor to amplify the inflammatory responses and it is known to be involved in chronic inflammatory diseases [52]. In our study, functional immune system process GO-terms “positive regulation of isotype switching to IgA isotype” was a bigger cluster among fourteen total clusters identified and it was considered an indication of chronic inflammation. However, the involvement of IgA in the chronic inflammatory diseases of mice needs further investigation due to the differences between the FC receptors of IgA and their functions in mice and humans [53,54,55].

Myeloid cells, also known as granulocytes including monocytes and erythrocytes, own a common ancestor, myeloid progenitors of hematopoietic stem cells. Transcription factors and colony-stimulating factors lead the commitment of myeloid cells to their final differentiation lineage. Myeloid cells are responsible for innate immunity and are normally recruited into the area of damaged tissues where they perform phagocytosis and release inflammatory cytokines, such as IL-1, IL-6, TNF-α, and chemokines, to amplify the inflammatory responses [56]. Moreover, myeloid cells are responsible for initiating adaptive immune responses involving lymphocytes, T cells, and B cells [57]. Enrichment of the myeloid cell differentiation pathway was significant in our study to support the existence of inflammatory responses in HtrA1 Tg mice. In addition, we found indicators of enrichment of adaptive immunity pathways: 1. Peptide antigen assembly with MHC class I protein complex, 2. Regulation of T cell mediated immunity, and 3. Regulation of activated T cell proliferation; and innate immunity: 1. Monocyte chemotaxis, 2. Granulocyte differentiation. All in all, RNA-seq analysis of the eyecup of Htra1 mice revealed its angiogenic and inflammatory phenotype.

### 3.7. Limitations

A limitation of this study was mainly the difference in the immune response between humans and mice. For example, in an acute phase inflammation, a massive influx of macrophages is not observed in human AMD [58]. Here, we detected macrophages only on flatmounts but not in the specimen sections, which will allow the detection of a small number of macrophages. Whether circulating monocytes in the CAG-HtrA1 Tg mice might have invaded the Bruch’s membrane, as demonstrated in a previous study, needs further investigation [14]. In addition, it is possible that the cytokines produced by the activated macrophages are involved in the recruitment of monocytes [59,60]. Our results were partially concordant with the results from the HtrA1 transgenic mice using the RPE-specific promoter, in which severe PCV exhibited prominent immune complex deposition, complement activation, and infiltration of inflammatory cells [11].

## 4. Materials and Methods

### 4.1. Animals

The development of the mouse line we used in the current study was described elsewhere [13]. Transgenic mice used in the study were offspring from haplodeficient heterozygous breeding pairs. Wild-type littermates were used as control animals. All animal procedures were reviewed and approved by the SingHealth Institutional Animal Care and Use Committee (IACUC#: 2018/SHS/1405) and the current study adhered to the ARVO statement for the use of animals in Ophthalmic and Vision Research. Mice were housed in standard cages (5 mice per cage), with free access to food and water under a 12 h light and 12 h dark cycle with a temperature-controlled environment. We performed genotyping for CAG HtrA1 Tg mice by PCR of genomic DNA extracted from tail snips using GoTaq(R) G2 Hot Start Green Master Mix (Promega Pte Ltd., Madison, WI, USA). Forward and reversed primers used for genotyping were ACTTCCTTTGTCCCAAATCTGT and AGCAATAAAGTTGTACTTATGACGCAAA, respectively.

### 4.2. CFP, OCT and FFA

For all the in vivo imaging experiments, the mice were anaesthetized by intraperitoneal (IP) injection of a mixture of Ketamine (20 mg/kg) and Xylazine (2 mg/kg). The pupil was dilated by adding 1 drop of 1% tropicamide and 2.5% phenylephrine (Alcon Laboratories, Inc., Fort Worth, TX, USA). The ophthalmic gel was used to keep the mouse cornea moist and prevent it from drying out. Funduscopic examinations and optical coherence tomography (OCT) images were performed by using Micron IV rodent comprehensive system from Phoenix Research Laboratories (San Ramon, CA, USA). For fundus fluorescein angiography (FFA), 10% sodium fluorescein dye (0.01 mL per 5 to 6 g body weight), diluted in sterile saline was administered via IP and the resulting images were captured from MICRON IV with a built-in filter for fluorescence, immediately after three to five minutes of fluorescein dye injection.

### 4.3. OCTA and ICGA Imaging

Mice OCTA images were obtained from the PLEX Elite 9000 (Carl Zeiss Meditec, Inc., Dublin, CA, USA); a wide field en face swept source OCT (SS-OCT) imaging system with a tuneable laser for the assessment of the retinal and choroidal vasculature. It was scanned in a central wavelength, λc = 1050 nm; and bandwidth, λ = 100 nm, using a wavelength scanning laser, and images were acquired using the PLEX Elite 9000 scanner. The system operation speed was 100,000 A scan/second. The OCT sessions were performed with an ultra-wide field of view at 56°. The frame rate was 100 khz and the axial resolution was 6 µm [61]. Polymethyl methacrylate (PMMA) contact lenses (Diameter: 3.2 mm, Cornea surfaces R 1.7 mm, outer surfaces R 1.8 mm, central thickness 0.3 mm—Heidelberg Engineering (Heidelberg, Germany)) were used to mount the mouse eyes as it prevents corneal dehydration, cataract formation during prolonged imaging sessions, and optimizes image clarity during OCTA imaging. To investigate the polypoidal lesions in the choroidal vasculature region, ICGA was performed with a Heidelberg Retina Angiograph (HRA)-OCT device (Spectralis) from Heidelberg Engineering (Heidelberg, Germany). Indocyanine green (2 mg/kg; Aschheim, Germany) was injected via the retro-orbital sinus route. We measured ICGA in 3 different stages, early phase (1–5 min after injection), middle phase (6–8 min after injection), and late phase (9–12 min after injection).

### 4.4. Histology and Immunohistochemistry

The whole eye was enucleated and embedded in the optimal cutting temperature liquid compound (Leica biosystems, Vista, CA, USA) at −80 °C. The cryoblocks were placed at −20 °C for at least 30 min prior to sectioning. The frozen sections were cut on a Micron HM 525 Cryostat (Thermo Fisher Scientific, Waltham, MA, USA) at 6µm thickness and were placed on microscopic glass slides (Polysine; Gerhard Menzel, Thermo Fisher Scientific, Newington, CT, USA). Sections were air-dried at room temperature (25 °C) for 1 h then stored at −80 °C. Before the immunohistochemical experiments, the specimen slides were left to warm at room temperature, and post-fixed with 4% paraformaldehyde for 5 min. Specimen slides were then blocked with 10% goat serum in PBS with 0.3% Triton X-100 for an hour at room temperature. The specimen slides were then incubated with an Anti-Apo lipoprotein E (ApoE) antibody (Goat polyclonal, 178479, Merck, MA, USA—# 3386966), Anti-CD31 antibody (Rabbit polyclonal, Abcam, ab124432, Cambridge, MA, USA—#GR324023), Ki67 antibody (Rabbit polyclonal, Abcam, ab15580, Cambridge, MA, USA), Recoverin antibody (Mouse monoclonal, ab31928, Cambridge, MA, USA), and VEGFR2 antibody (Rabbit polyclonal, ab39256, Cambridge, MA, USA) overnight at 4 °C. Phosphate-buffered saline (PBS) washes were performed before secondary antibody incubations for 1 h with goat anti-rabbit Alexa Fluor 568 secondary antibody and Donkey anti-goat Alexa Fluor 488 secondary antibody (1:1000 dilution, Molecular probe, Life Technologies, Carlsbad, CA, USA) in the dark. An additional three washes with PBS were performed prior to mounting using diamond antifade with 4′,6-diamidino-2-phenylindole (DAPI) (Life technologies, Eugene, OR, USA). The specimens on slides were scanned by a Zeiss Axio Imager Z1 Upright Trinocular Fluorescence Microscope (Oberkochen, Germany).

Haematoxylin and Eosin (H&E) staining of frozen sections was performed by staining of haematoxylin for 3 min followed by intensification of nucleus staining with Scott’s tap water for 5 min and counterstained with Eosin for 3 min. The frozen sections were then washed in tap water, mounted with paramount, and imaged under the bright field using the Nikon C2 system (Nikon Microscopy, Melville, NY, USA).

Flatmounts were stained with antibodies against endothelial cell, pericyte, and macrophage markers (CD31, NG2, and F4/80). For flat-mount preparations, mouse eyes were enucleated and post fixed with 4% paraformaldehyde for 2 min. The retina and the eyecup containing RPE-choroid complex were separated and dissected in a symmetrical flat flower shape in 2X PBS. The retina and the RPE-choroid containing eyecup were fixed again in absolute methanol and flattened before being stained with primary antibodies, including CD31 (rat monoclonal, 553370, BD Biosciences, San Jose, CA, USA, RRID: AB_394816), NG2 (rabbit polyclonal, AB5320, Merck Millipore, Germany, RRID: AB_11213678), and F4/80 (rat monoclonal, AbD Serotec) at 4 °C overnight. PBS washes were performed before secondary antibody incubations for 1 h with Alexa Fluor 488 or Alexa Fluor 594 secondary antibodies (A-11006 or A-11012, Thermo Fisher Scientific, USA) and DAPI. The choroid and retina flatmouts were mounted using Diamond Antifade Mountant and examined by confocal microscopy (Zeiss LSM 800, Zeiss, Germany). The retinal vasculature was analyzed using AngioTool.

### 4.5. Flow Cytometric Analysis of Monocytes

Cells were isolated from the retina and choroid as described previously [62]. The samples were analyzed using FACSAria III (BD Biosciences, San Jose, CA, USA) and FlowJo software (Tree Star, Ashland, OR, USA). Anti-F4/80-APC, anti-Ly6C-FITC, anti-Ly6G-APC-Cy7 (all from BioLegend, San Diego, CA, USA), and anti-CD11b-PE (eBioscience, San Diego, CA, USA) were used. Monocytes subsets were identified as CD11bhiF4/80hiLy6Chi/int/lo and can be subdivided into 3 subsets; classical, intermediate, and non-classical monocytes [63].

### 4.6. Laser-Induced CNV

This was performed using MERILAS 532α (MERIDIAN AG, Switzerland) attached to Micron IV (Phoenix lab, Bend, OR, USA) to induce laser spots surrounding the optic head in both eyes of the animals at 12, 3, 6, and 9 o’clock positions. Breakage of Bruch’s membrane was confirmed by a central bubble formation at the lasered area. Seven days after the photocoagulation, the lesions were imaged with FFA and quantified as reported previously [16,64]. Eight-week-old mice were used for protein concentration determination (*n* = 8 for each group). Ten laser photocoagulations were applied to one eye and the contralateral eye was used as controls. Three days after laser treatment, cell lysate from RPE/choroid was isolated using a lysis buffer. LUNARISTM multiplex protein analysis (Ayoxxa, Deutshland) was used for the quantification of VEGF, Ang1, and Ang2 levels. The experimental procedures were performed according to the technical manual.

### 4.7. Choroidal Sprouting Assay

Postnatal day 3 (P3) and adult mouse eyes were enucleated. Retina was separated from the RPE/Choroid/sclera complex. The RPE/Choroid/sclera complex was cut into 1 mm pieces in PBS before being cultured in the Matrigel with EGM2 medium (Lonza, Basel, Switzerland) of 37 °C with 5% CO_2_ for 48 h. The vessel outgrowth from the choroid explants was monitored daily and imaged using Zeiss axio observer z1 microscope. Microvascular sprouts were quantified using ImageJ (National Institute of Health).

### 4.8. RNA Extraction, Sequencing, and Data Analyses

Posterior eye cups (*n* = 8) from transgenic HtrA1 Tg mice (5~12 months) were collected as well as an equal number of age-matched WT mice. Briefly, mice were anaesthetized by using a mixture of appropriate dosage of ketamine and xylazine, as mentioned elsewhere, followed by cervical dislocation. Enucleated eyes were collected into ice-cold PBS followed by the removal of the smooth muscles around the eyeball and anterior parts of the eye cups such as the cornea, iris, lens, vitreous, and retina. The optic nerve was also trimmed as close as possible to the eyeball. Posterior parts of the whole eye cup, from the retina and RPE layer towards the sclera and including the neural retina region, were transferred into a homogenizer tube (2 mL), preloaded with 1 mL of ice-cold TRI reagent (Zymoresearch, CA, USA) and a 5 mm stainless steel bead (Qiagen, Germany). Homogenization was conducted at 25 frequency/second for 5 min, 2 times using a TissueLyser II (Qiagen, Germany) followed by centrifugation of the homogenizer tube at 200 g for 3 min. Supernatant was transferred into a total RNA extraction column and processed further according to the manufacturer’s instruction (RNA MicroPrep, Zymoresearch, Irvine, CA, USA). The extracted RNA concentration and purity were checked by Nanodrop at wavelengths of 260 nm and 280 nm (NanoDrop~ND-3300 Fluorospectrometer). RNA-seq was performed by Illumina Novaseq 6000, paired-end 2 × 150 bp at Novogene (Beijing, China). RNA-seq data was analyzed by using Galaxy RNA-seq analysis software (Baltimore, MD, USA) for differentially expressed genes (DEGs) followed by Cytoscape software version 3.9.1 (ISB, Seattle, WA, USA) with ClueGo (version 2.5.9) for Reactome, KEGG pathways enrichments as well as immune system process of Gene Ontology.

### 4.9. Statistical Analyses

IBM SPSS v. 20.0 software (IBM Corp., Armonk, NY, USA) was utilized for statistical analysis. Chi-square test was used for the comparison of the prevalence of subretinal lesions between the CAG-HtrA1 Tg and WT mice and the Mann–Whitney U test was used to compare the differences of monocyte subpopulation, leakage from the laser induced CNV, area of choroidal sprouting between the CAG-HtrA1 Tg, and WT mice. Significance level was defined as *p* < 0.05.

## 5. Conclusions

In summary, overexpressed HtrA1 could mediate retinal pathology, resulting in the accumulation of macrophage infiltration and increased RPE migration that links to the angiogenic pathway. Furthermore, pathway enrichment analyses of DEGs showed angiogenic and inflammatory phenotypes of HtrA1 Tg mice. The main theme of the current study is to document the phenotypical and morphological changes of the CAG-HtrA1 Tg mice. Future studies should be directed towards understanding the molecular functions of HtrA1 in the pathogenesis of retinochoroidal diseases.

## Figures and Tables

**Table 1:** Summary of the current study findings in comparisons between Tg and WT mice.

Changes	HtrA1 Tg	WT
**Lesion specific**		
Type 1—Large white deposits	Present on fundus examination in 33% (20/60) CFP: 0.2–0.5-disc diameter OCT: Hyper-reflectivity in outer retina and localized subretinal fluid FFA: no leakage OCTA: no flow signal	Present on fundus examination in 7% (1/15)
Type 2- multiple small white-yellowish foci	Not discernible on cross-sectional OCT, but clearly visible on avascular slab on en face OCT. FFA: no leakage OCTA: no flow signal	Same
**Global**		
ICGA	No polypoidal vascular dilatation observed Multiple small punctate hyperfluorescent spots on ICGA [40%~(4/10)] and en face OCT	No hyperfluorescent dots observed (*n* = 3)
OCTA	No neovascularization (*n* = 15)	No neovascularization (*n* = 6)
IHC	Positive for both ApoE and CD31 (*n* = 4)	Positive for both ApoE and CD31 (*n* = 4)

FFA: fundus fluorescence angiography; ICGA: indocyanine green angiography; IHC: immunohisotochemistry; OCT: Optical coherence tomography; OCTA: optical coherence tomography angiography.

## Data Availability

The data that support the findings of this study are available from the corresponding author upon pragmatic request.

## References

[1] Wong W.L., Su X., Li X., Cheung C.M.G., Klein R., Cheng C.Y., Wong T.Y. (2014). Global prevalence of age-related macular degeneration and disease burden projection for 2020 and 2040: A systematic review and meta-analysis. Lancet Glob. Health.

[2] Yannuzzi L.A., Ciardella A., Spaide R.F., Rabb M., Freund K.B., Orlock D.A. (1997). The Expanding Clinical Spectrum of Idiopathic Polypoidal Choroidal Vasculopathy. Arch. Ophthalmol..

[3] Imamura Y., Engelbert M., Iida T., Freund K.B., Yannuzzi L.A. (2010). Polypoidal Choroidal Vasculopathy: A Review. Surv. Ophthalmol..

[4] Lima L.H., Schubert C., Ferrara D.C., Merriam J.E., Imamura Y., Freund K.B., Spaide R.F., Yannuzzi L.A., Allikmets R. (2010). Three Major Loci Involved in Age-Related Macular Degeneration Are Also Associated with Polypoidal Choroidal Vasculopathy. Ophthalmology.

[5] Lee K.Y., Vithana E.N., Mathur R., Yong V.H., Yeo I.Y., Thalamuthu A., Lee M.-W., Koh A.H., Lim M.C., How A.C. (2008). Association Analysis of *CFH*, *C2*, *BF*, and *HTRA1* Gene Polymorphisms in Chinese Patients with Polypoidal Choroidal Vasculopathy. Investig. Opthalmol. Vis. Sci..

[6] DeWan A., Liu M., Hartman S., Zhang S.S.-M., Liu D.T.L., Zhao C., Tam P.O.S., Chan W.M., Lam D.S.C., Snyder M. (2006). *HTRA1* Promoter Polymorphism in Wet Age-Related Macular Degeneration. Science.

[7] Jones A., Kumar S., Zhang N., Tong Z., Yang J.-H., Watt C., Anderson J., Amrita, Fillerup H., McCloskey M. (2011). Increased expression of multifunctional serine protease, HTRA1, in retinal pigment epithelium induces polypoidal choroidal vasculopathy in mice. Proc. Natl. Acad. Sci. USA.

[8] Hageman G.S., Anderson D.H., Johnson L.V., Hancox L.S., Taiber A.J., Hardisty L.I., Hageman J.L., Stockman H.A., Borchardt J.D., Gehrs K.M. (2005). A common haplotype in the complement regulatory gene factor H (HF1/CFH) predisposes individuals to age-related macular degeneration. Proc. Natl. Acad. Sci. USA.

[9] Pan Y., Iejima D., Nakayama M., Suga A., Noda T., Kaur I., Das T., Chakrabarti S., Guymer R.H., DeAngelis M.M. (2021). Binding of Gtf2i-β/δ transcription factors to the ARMS2 gene leads to increased circulating HTRA1 in AMD patients and in vitro. J. Biol. Chem..

[10] Kumar S., Berriochoa Z., Ambati B.K., Fu Y. (2014). Angiographic Features of Transgenic Mice With Increased Expression of Human Serine Protease HTRA1 in Retinal Pigment Epithelium. Investig. Opthalmol. Vis. Sci..

[11] Kumar S., Nakashizuka H., Jones A., Lambert A., Zhao X., Shen M., Parker M., Wang S., Berriochoa Z., Fnu A. (2017). Proteolytic Degradation and Inflammation Play Critical Roles in Polypoidal Choroidal Vasculopathy. Am. J. Pathol..

[12] Kumar S., Berriochoa Z., Jones A.D., Fu Y. (2014). Detecting Abnormalities in Choroidal Vasculature in a Mouse Model of Age-related Macular Degeneration by Time-course Indocyanine Green Angiography. J. Vis. Exp..

[13] Nakayama M., Iejima D., Akahori M., Kamei J., Goto A., Iwata T. (2014). Overexpression of *HtrA1* and Exposure to Mainstream Cigarette Smoke Leads to Choroidal Neovascularization and Subretinal Deposits in Aged Mice. Investig. Opthalmol. Vis. Sci..

[14] Yanagi Y., Foo V.H.X., Yoshida A. (2018). Asian age-related macular degeneration: From basic science research perspective. Eye.

[15] Meyer J.H., Larsen P.P., Strack C., Harmening W.M., Krohne T.U., Holz F.G., Schmitz-Valckenberg S. (2019). Optical coherence tomography angiography (OCT-A) in an animal model of laser-induced choroidal neovascularization. Exp. Eye Res..

[16] Agrawal R., Tun S.B.B., Balne P.K., Zhu H.-Y., Khandelwal N., A Barathi V. (2018). Fluorescein Labeled Leukocytes for *in vivo* Imaging of Retinal Vascular Inflammation and Infiltrating Leukocytes in Laser-Induced Choroidal Neovascularization Model. Ocul. Immunol. Inflamm..

[17] Lu Z., Lin V., May A., Che B., Xiao X., Shaw D.H., Su F., Wang Z., Du H., Shaw P.X. (2019). HTRA1 synergizes with oxidized phospholipids in promoting inflammation and macrophage infiltration essential for ocular VEGF expression. PLoS ONE.

[18] Yannuzzi L.A. (2011). Indocyanine Green Angiography: A Perspective on Use in the Clinical Setting. Am. J. Ophthalmol..

[19] Kim S.-J., Kim J.-S., Papadopoulos J., Kim S.W., Maya M., Zhang F., He J., Fan D., Langley R., Fidler I.J. (2009). Circulating Monocytes Expressing CD31: Implications for Acute and Chronic Angiogenesis. Am. J. Pathol..

[20] Shao Z., Friedlander M., Hurst C.G., Cui Z., Pei D.T., Evans L.P., Juan A., Tahiri H., Duhamel F., Chen J. (2013). Choroid Sprouting Assay: An Ex Vivo Model of Microvascular Angiogenesis. PLoS ONE.

[21] Hsu S.T., Vajzovic L. (2019). Analyzing Optical Coherence Tomography Angiography. Handbook of Pediatric Retinal OCT and the Eye-Brain Connection.

[22] Caplash S., Kodati S., Cheng S.K., Akanda M., Vitale S., Thompson I., Gangaputra S., Sen H.N. (2019). Repeatability of Optical Coherence Tomography Angiography in Uveitic Eyes. Transl. Vis. Sci. Technol..

[23] Stanzel T.P., Devarajan K., Lwin N.C., Yam G.H., Schmetterer L., Mehta J.S., Ang M. (2018). Comparison of Optical Coherence Tomography Angiography to Indocyanine Green Angiography and Slit Lamp Photography for Corneal Vascularization in an Animal Model. Sci. Rep..

[24] Cheung C.M.G., Lee W.K., Koizumi H., Dansingani K., Lai T.Y.Y., Freund K.B. (2019). Pachychoroid disease. Eye.

[25] Matsumoto H., Mukai R., Morimoto M., Tokui S., Kishi S., Akiyama H. (2019). Clinical characteristics of pachydrusen in central serous chorioretinopathy. Graefe’s Arch. Clin. Exp. Ophthalmol..

[26] Tsujikawa A., Ojima Y., Yamashiro K., Ooto S., Tamura H., Nakagawa S., Yoshimura N. (2010). Punctate hyperfluorescent spots associated with central serous chorioretinopathy as seen on indocyanine green angiography. Retina.

[27] Jun S., Datta S., Wang L., Pegany R., Cano M., Handa J.T. (2018). The impact of lipids, lipid oxidation, and inflammation on AMD, and the potential role of miRNAs on lipid metabolism in the RPE. Exp. Eye Res..

[28] Tan C.S., Ngo W.K., Lim L.W., Tan N.W., Lim T.H., on behalf of the EVEREST Study Group (2016). EVEREST study report 3: Diagnostic challenges of polypoidal choroidal vasculopathy. Lessons learnt from screening failures in the EVEREST study. Graefe’s Arch. Clin. Exp. Ophthalmol..

[29] Mishima H., Kondo K. (1981). Extrusion of lysosomal bodies from apical mouse retinal pigment epithelium. Albr. Graefes Arch. Klin. Exp. Ophthalmol..

[30] Xu H., Chen M., Manivannan A., Lois N., Forrester J.V. (2008). Age-dependent accumulation of lipofuscin in perivascular and subretinal microglia in experimental mice. Aging Cell.

[31] Tamiya S., Liu L., Kaplan H.J. (2010). Epithelial-Mesenchymal Transition and Proliferation of Retinal Pigment Epithelial Cells Initiated upon Loss of Cell-Cell Contact. Investig. Opthalmol. Vis. Sci..

[32] Curcio C.A., Zanzottera E.C., Ach T., Balaratnasingam C., Freund K.B. (2017). Activated Retinal Pigment Epithelium, an Optical Coherence Tomography Biomarker for Progression in Age-Related Macular Degeneration. Investig. Opthalmol. Vis. Sci..

[33] Nassisi M., Lei J., Abdelfattah N., Karamat A., Balasubramanian S., Fan W., Uji A., Marion K.M., Baker K., Huang X. (2019). OCT Risk Factors for Development of Late Age-Related Macular Degeneration in the Fellow Eyes of Patients Enrolled in the HARBOR Study. Ophthalmology.

[34] Curcio C.A., Johnson M., Huang J.-D., Rudolf M. (2009). Aging, age-related macular degeneration, and the response-to-retention of apolipoprotein B-containing lipoproteins. Prog. Retin. Eye Res..

[35] Hageman G.S. (2001). An Integrated Hypothesis That Considers Drusen as Biomarkers of Immune-Mediated Processes at the RPE-Bruch’s Membrane Interface in Aging and Age-Related Macular Degeneration. Prog. Retin. Eye Res..

[36] Zhou J., Chen F., Yan A., Xia X. (2021). Overexpression of HTRA1 increases the proliferation and migration of retinal pigment epithelium. Adv. Clin. Exp. Med..

[37] Combadiere C., Feumi C., Raoul W., Keller N., Rodero M., Pézard A., Lavalette S., Houssier M., Jonet L., Picard E. (2007). CX3CR1-dependent subretinal microglia cell accumulation is associated with cardinal features of age-related macular degeneration. J. Clin. Investig..

[38] Luhmann U.F.O., Robbie S., Munro P.M.G., Barker S.E., Duran Y., Luong V., Fitzke F.W., Bainbridge J.W.B., Ali R.R., MacLaren R.E. (2009). The Drusenlike Phenotype in Aging *Ccl2*-Knockout Mice Is Caused by an Accelerated Accumulation of Swollen Autofluorescent Subretinal Macrophages. Investig. Opthalmol. Vis. Sci..

[39] Joussen A.M., Ricci F., Paris L.P., Korn C., Quezada-Ruiz C., Zarbin M. (2021). Angiopoietin/Tie_2_ signalling and its role in retinal and choroidal vascular diseases: A review of preclinical data. Eye.

[40] Owen L.A., Shirer K., Collazo S.A., Szczotka K., Baker S., Wood B., Carroll L., Haaland B., Iwata T., Katikaneni L.D. (2020). The Serine Protease HTRA-1 Is a Biomarker for ROP and Mediates Retinal Neovascularization. Front. Mol. Neurosci..

[41] Feng Y., Zhang T., Wang Y., Xie M., Ji X., Luo X., Huang W., Xia L. (2021). Homeobox Genes in Cancers: From Carcinogenesis to Recent Therapeutic Intervention. Front. Oncol..

[42] McGinnis W., Levine M.S., Hafen E., Kuroiwa A., Gehring W.J. (1984). A conserved DNA sequence in homoeotic genes of the Drosophila Antennapedia and bithorax complexes. Nature.

[43] Park H., Choi H.-J., Kim J., Kim M., Rho S.-S., Hwang D., Kim Y.-M., Kwon Y.-G. (2011). Homeobox D1 regulates angiogenic functions of endothelial cells via integrin β1 expression. Biochem. Biophys. Res. Commun..

[44] Toshner M., Dunmore B.J., McKinney E.F., Southwood M., Caruso P., Upton P.D., Waters J.P., Ormiston M.L., Skepper J.N., Nash G. (2014). Transcript analysis reveals a specific HOX signature associated with positional identity of human endothelial cells. PLoS ONE.

[45] Hansen S.L., Myers C.A., Charboneau A., Young D.M., Boudreau N. (2003). HoxD3 Accelerates Wound Healing in Diabetic Mice. Am. J. Pathol..

[46] Boudreau N., Andrews C., Srebrow A., Ravanpay A., Cheresh D.A. (1997). Induction of the Angiogenic Phenotype by Hox D3. J. Cell Biol..

[47] Chen Y., Xu B., Arderiu G., Hashimoto T., Young W.L., Boudreau N., Yang G.-Y. (2004). Retroviral Delivery of Homeobox D3 Gene Induces Cerebral Angiogenesis in Mice. J. Cereb. Blood Flow Metab..

[48] Barcia C., Bautista V., Sanchez-Bahillo A., Fernandez-Villalba E., Faucheux B., Poza M.P.Y., Barreiro A.F., Hirsch E., Herrero M.-T. (2005). Changes in vascularization in substantia nigra pars compacta of monkeys rendered parkinsonian. J. Neural Transm..

[49] Bradaric B.D., Patel A., Schneider J.A., Carvey P.M., Hendey B. (2011). Evidence for angiogenesis in Parkinson’s disease, incidental Lewy body disease, and progressive supranuclear palsy. J. Neural Transm..

[50] Faucheux B.A., Agid Y., Hirsch E., Bonnet A.-M. (1999). Blood vessels change in the mesencephalon of patients with Parkinson’s disease. Lancet.

[51] Yu H., Yuan L., Yang Y., Ma S., Peng L., Wang Y., Zhang C., Li T. (2016). Increased serum IgA concentration and plasmablast frequency in patients with age-related macular degeneration. Immunobiology.

[52] Hansen I.S., Baeten D.L.P., Den Dunnen J. (2019). The inflammatory function of human IgA. Cell. Mol. Life Sci..

[53] Mestas J., Hughes C.C.W. (2004). Of mice and not men: Differences between mouse and human immunology. J. Immunol..

[54] Bruhns P., Jönsson F. (2015). Mouse and human FcR effector functions. Immunol. Rev..

[55] Cerutti A. (2008). The regulation of IgA class switching. Nat. Rev. Immunol..

[56] Kawamoto H., Minato N. (2004). Myeloid cells. Int. J. Biochem. Cell Biol..

[57] Smith N.C., Rise M.L., Christian S.L. (2019). A Comparison of the Innate and Adaptive Immune Systems in Cartilaginous Fish, Ray-Finned Fish, and Lobe-Finned Fish. Front. Immunol..

[58] Vogt S.D., Curcio C.A., Wang L., Li C.-M., McGwin G., Medeiros N.E., Philp N.J., Kimble J.A., Read R.W. (2011). Retinal pigment epithelial expression of complement regulator CD46 is altered early in the course of geographic atrophy. Exp. Eye Res..

[59] Sica A., Erreni M., Allavena P., Porta C. (2015). Macrophage polarization in pathology. Exp..

[60] Mantovani A., Sica A., Sozzani S., Allavena P., Vecchi A., Locati M. (2004). The chemokine system in diverse forms of macrophage activation and polarization. Trends Immunol..

[61] Tan B., Barathi V.A., Lin E., Ho C., Gan A., Yao X., Chan A., Wong D.W., Chua J., Tan G.S. (2020). Longitudinal Structural and Microvascular Observation in RCS Rat Eyes Using Optical Coherence Tomography Angiography. Investig. Opthalmol. Vis. Sci..

[62] Tan X., Fujiu K., Manabe I., Nishida J., Yamagishi R., Nagai R., Yanagi Y. (2015). Choroidal neovascularization is inhibited via an intraocular decrease of inflammatory cells in mice lacking complement component C3. Sci. Rep..

[63] Fujiu K., Manabe I., Nagai R. (2011). Renal collecting duct epithelial cells regulate inflammation in tubulointerstitial damage in mice. J. Clin. Investig..

[64] Tobe T., Ortega S., Luna J.D., Ozaki H., Okamoto N., Derevjanik N.L., Vinores S.A., Basilico C., Campochiaro P.A. (1998). Targeted Disruption of the FGF2 Gene Does Not Prevent Choroidal Neovascularization in a Murine Model. Am. J. Pathol..

